# Organic Cation Transporters (OCTs) in EpiAirway™, a Cellular Model of Normal Human Bronchial Epithelium

**DOI:** 10.3390/biomedicines8050127

**Published:** 2020-05-19

**Authors:** Amelia Barilli, Rossana Visigalli, Francesca Ferrari, Maria Di Lascia, Benedetta Riccardi, Paola Puccini, Valeria Dall’Asta, Bianca Maria Rotoli

**Affiliations:** 1 Laboratory of General Pathology, Department of Medicine and Surgery, University of Parma, 43125 Parma, Italy; amelia.barilli@unipr.it (A.B.); rossana.visigalli@unipr.it (R.V.); francesca.ferrari@unipr.it (F.F.); biancamaria.rotoli@unipr.it (B.M.R.); 2 Preclinical Pharmacokinetics, Biochemistry & Metabolism Department, Chiesi Farmaceutici, 43122 Parma, Italy; M.dilascia@chiesi.com (M.D.L.); B.Riccardi@chiesi.com (B.R.); P.Puccini@chiesi.com (P.P.)

**Keywords:** bronchial epithelium, organic cation transporters, pulmonary drug delivery, *SLC22A1* (OCT1), *SLC22A3* (OCT3), *SLC6A14* (ATB^0,+^)

## Abstract

Organic cation transporters (OCTs) and novel organic cation transporters (OCTNs) are responsible for drug delivery in the intestine and kidney; in the lung, OCTs mediate inhaled drugs’ transport, although their physiological role in airways remains poorly understood. The studies addressing OCTs/OCTNs in human airways were mostly performed in immortal or transformed cell lines; here, we studied OCTs in EpiAirway™, a recently developed in vitro model of normal bronchial epithelium. Calu-3 monolayers were used for comparison. The activity of OCTs was evaluated by measuring the uptake of 1-methyl-4-phenylpyridinium (MPP^+^) at the apical and basolateral side of monolayers and protein expression through Western Blot analysis. OCTs and OCTNs expression, along with that of Amino acid Transporter B^0,+^ (ATB^0,+^)transporter, was determined by measuring the number of mRNA molecules through quantitative Polymerase Chain Reaction (qPCR). The interaction of the transporters with bronchodilators was also assessed. Results highlight significant differences between Calu-3 cells and EpiAirway™, since, in the latter, OCTs are active only on the basolateral membrane where they interact with the bronchodilator ipratropium. No activity of OCTs is detectable at the apical side; there, the most abundant carrier is, instead, *SLC6A14*/ATB^0,+^, that can thus be potentially listed among organic cation transporters responsible for drug delivery in the lung.

## 1. Introduction

Human transporters belonging to the solute carrier family 22A (SLC22A) play a central role in physiology, pharmacology, and toxicology because of the broad spectrum of endogenous metabolites, drugs, and toxins that they can move across cell plasma membranes [1,2].

According to a simplified classification based on the electric nature of the substrates, the human SLC22A family includes Organic cation transporters (OCTs: OCT1, OCT2, and OCT3) that operate as electrogenic uniporters for organic cations, the so-called “Novel” organic cation transporters (OCTNs) mediating Na^+^-cotransport of selected zwitterions, and Organic Anions Transporters (OATs), that physiologically work as organic anion exchangers [3]. For the transepithelial secretion of organic cations, OCTs are frequently paired to the obligatory exchangers multidrug and toxin extruders (MATEs) belonging to the SLC47 family [4], with OCTs typically operating the basolateral uptake of organic cations and MATEs responsible for the apical efflux [5,6].

All three OCTs transport endogenous compounds, such as monoamine neurotransmitters, carnitine derivatives, and creatinine, as well as several drugs, and model substrates for OCTs are 1-methyl-4-phenylpyridinium (MPP^+^) and tetraethylammonium (TEA) [3,7]. These transporters display a characteristic multi-selectivity with broadly overlapping sites of expression in many tissues such as liver, kidney, heart, skeletal muscle, placenta, lung, brain, immune system [8,9], as well as in the whole gastrointestinal tract [10]. Studies concerning OCTs transporters have mainly focused on hepatocytes and kidney proximal tubule, owing to the key role of these tissues in the metabolism of endogenous compounds and xenobiotics and in the excretion of water-soluble drugs and derivatives [5]. As far as the lung is concerned, the physiological role of OCT transporters is thus far incompletely understood, despite the fact that it is known that OCTs mediate the transport of inhaled drugs [11,12]. The expression and activity of OCTs has been addressed in various cell models representative of different respiratory tracts [13,14,15]. To this concern, in a previous study, we focused on OCTs in respiratory epithelial cell lines of human origin, i.e., in Calu-3, 16HBE14o-, NCl-H441, and BEAS-2B [16], and highlighted significant differences in the expression of the transporters among the cell models. Indeed, while A549 and NCl-H441 were endowed with the activity of the sole OCT3 and OCT1 respectively, both transporters were operative in Calu-3 and BEAS-2B. OCT2 transporter was not detected in any of the cell lines employed. The major concern raised from that and similar studies is that they all employed transfected or immortal cell lines as models, and the possibility exists that their biological features may differ from those of primary differentiated cells. Thus, the need for a reliable model of normal respiratory epithelium in vitro is urgent for research concerning drug absorption and disposition in the airways.

Recently, innovative culture systems of human respiratory and nasal epithelial cells, such as the EpiAirway™ (MatTek Corporation) and MucilAir™ (Epithelix) systems, have been developed [6]. Due to their composition and structure, these models, composed of well-differentiated ciliated and goblet cells, properly reflect the phenotype of barriers in vivo [17], thus appearing useful tools for studies of drug permeability. Since no information is available about OCTs in this cell system, the aim of the present study is to characterize their expression and activity in EpiAirway™. 

## 2. Materials and Methods

### 2.1. Cell Cultures

EpiAirway™ tissues (AIR-200-PE6.5), supplied by MatTek Lifesciences (Ashland, MA, USA), were used. Cultured on microporous membrane inserts at the air–liquid interface (ALI), EpiAirway™ recapitulates aspects of the in vivo microenvironment of the lung. This system is, indeed, produced from primary human tracheal-bronchial epithelial cells that form a fully differentiated, pseudostratified columnar epithelium containing mucus-producing goblet cells, ciliated cells, and basal cells. Upon arrival, tissue inserts were transferred to 24-well plates containing 600 μL of the AIR 200-M125 medium and equilibrated overnight at 37 °C and 5% CO_2_. Medium at the basolateral side was, then, renewed every day, while apical washes for mucus removal were performed employing the solution provided by the manufacturer. Cultures from five different healthy donors were employed.

Calu-3 cells (American Type Culture Collection), obtained from a human lung adenocarcinoma and derived from serous cells of proximal bronchial airways, were cultured in Eagle’s Minimum Essential Medium (EMEM) supplemented with 10% fetal bovine serum (FBS), sodium pyruvate (1 mM), and 1% penicillin/streptomycin. Cells between passages 25–30 were routinely cultured in 10 cm diameter dishes. For the experiments, Calu-3 cell monolayers were grown for 21 days at ALI. To this end, 10^5^ cells were seeded onto each Transwell polyester insert (0.33 cm^2^, 0.4 μm pore size; Falcon) and the apical medium was removed 24 h after seeding, while basolateral medium was renewed every other day. 

The integrity of cell monolayers for both EpiAirway™ and Calu-3 cells was verified by measuring the trans-epithelial electrical resistance (TEER) with an epithelial voltmeter (EVOM, World Precision Instruments). Cell cultures were employed when TEER values > 350 Ω∙cm^2^.

Where indicated, lipopolysaccharides from *E. coli* 0111:B4 (LPS) or tumor necrosis factor α (TNFα) were added in complete growth medium for 24 h.

### 2.2. MPP^+^ Uptake

MPP^+^ transport was measured both at the apical and the basolateral side of the monolayers. After two washes in pre-warmed transport buffer (Earle’s Balanced Salt Solution (EBSS) containing (in mM) 117 NaCl, 1.8 CaCl_2_, 5.3 KCl, 0.9 NaH_2_PO_4_, 0.8 MgSO_4_, 5.5 glucose, 26 Tris/HCl, adjusted to pH 7.4), cells were incubated in fresh transport buffer (100 μL in the apical and 600 μL in the basolateral compartment) containing l-[^3^H]MPP^+^ (50 µM, 2 μCi/mL). Where indicated, the inhibitors or the drugs were added to the transport buffer at the indicated concentrations. For a sodium-free EBSS, 117 mM NaCl was replaced with equimolar *N*-methyl-d-glucamine chloride. At the times indicated, transport buffer was removed, and the experiment was terminated by two rapid washes (<10 s) in ice-cold urea (300 mM). The filter was then detached from the insert and the ethanol soluble pool was extracted from monolayers. Radioactivity in cell extracts was determined with MicroBeta^2^ liquid scintillation spectrometer (Perkin Elmer, Milano, MI, Italy). In order to discriminate the contribution of OCT1 and OCT3 transporters, MPP^+^ uptake was measured both in the absence and in the presence of quinidine or corticosterone employed as preferential inhibitors of OCT1 [8,18,19] and OCT3 [5,20], respectively. Protein content in monolayers was determined directly in the filters using a modified Lowry procedure [21]. MPP^+^ uptake is expressed as µmoles/mL of intracellular water. To this end, cell volume was estimated from the distribution space of [^14^C]urea, as already described [22]. Briefly, EpiAirway™ and Calu-3 cells were incubated for 15 min, at both apical and basolateral side, in EBSS containing [^14^C]urea (0.5 mM; 2 μCi/mL). The incubations were terminated by two rapid washes (<10 s) in ice-cold 300 mM urea and radioactivity in cell extracts was determined as described above. Calculated cell volumes corresponded to 5.2 ± 0.12 (for EpiAirway™) and 5.4 ± 0.6 (for Calu-3) μL/mg of protein (not shown).

### 2.3. RT-qPCR Analysis

mRNA expression has been analysed through RT-qPCR, as previously described [23]. 1 µg of total RNA was reverse transcribed with RevertAid First Strand cDNA Synthesis Kit (Thermo Fisher Scientific, Monza, MB, Italy) and qPCR was performed on 20 ng of cDNA by employing the StepOnePlus Real-Time PCR System (Thermo Fisher Scientific). The amount of the genes of interest and of the reference gene RPL15 (ribosomal protein-like 15, NM_001253379.2) was monitored using specific forward/reverse primer pairs (Table 1) and SYBR™ Green PCR Master Mix (Thermo Fisher Scientific) and calculated relatively to that of the reference gene using the formula $1000\times 2^{∆Ct}$ (where $Ct=Ct_{RPL15}-Ct_{gene\;of\;interest}$). When required, the absolute quantification of mRNA molecules was performed as described previously [24]. cDNA obtained from human renal proximal tubular epithelial cells (HRPTEpC) and A549 alveolar cells was employed as a control for the analysis of SLC22A2/OCT2 and SLC47A1/MATE1 expression, respectively.

### 2.4. Western Blot Analysis

To monitor the expression of transporter proteins, cell monolayers were washed with ice-cold PBS and lysed in Laemmli Sample Buffer (62.5 M Tris-HCl, pH 6.8, 2% sodium dodecyl sulfate (SDS), 20% glycerol, 0.2 M dithiothreitol (DTT)). Western Blot analysis was then performed as previously described [25], by employing anti-OCT1 antibody (ABCAM, Prodotti Gianni, Milano, MI, Italy) (1:1000; Cat# ab181022) and anti-OCT3 by Sigma-Aldrich (1:2000; Cat# SAB4502815). Anti-α-tubulin by Sigma-Aldrich (1:1000; Cat# T5168) was employed as internal loading control. Horseradish peroxidase (HRP)-conjugated secondary antibodies (anti-rabbit and anti-mouse IgG) were provided by Cell Signaling Technology (Euroclone, Pero, MI, Italy; 1:10000). Immunoreactivity was visualized with Immobilon Western Chemiluminescent HRP Substrate (Merck, Milano, MI, Italy). Western Blot images were captured with iBright FL1500 Imaging System (Thermo Fisher) and analyzed with iBright Analysis Software.

### 2.5. Statistical Analysis

The statistical analysis was performed using GraphPad Prism 7 (GraphPad Software). All data were analyzed with a two tailed Student’s *t*-test for unpaired data.

### 2.6. Materials

*N*-Methyl-4-phenylpyridinium acetate-[*N*-methyl-^3^H] (MPP^+^), 81.3 Ci/mmol, and [^14^C]-Urea, 55.42 mCi/mmol, were obtained from Perkin Elmer. All other chemical and reagents were from Sigma-Aldrich. 

## 3. Results

**Figure 1:**
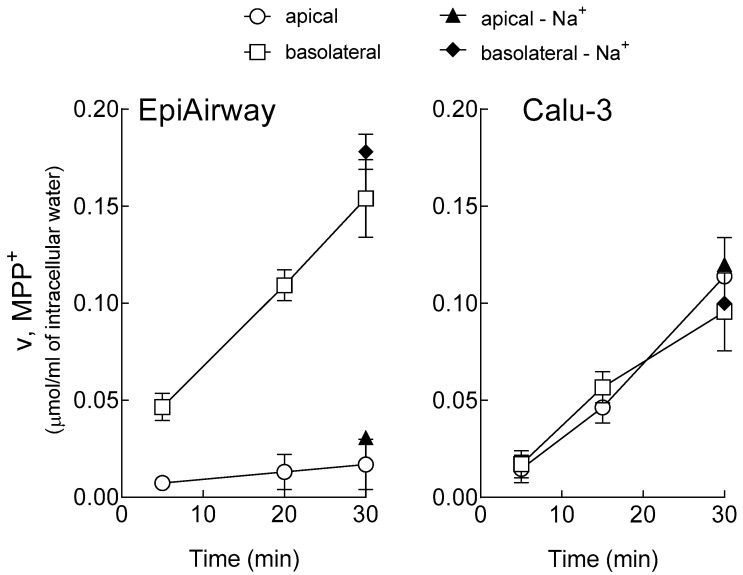
Time-dependent accumulation of 1-methyl-4-phenylpyridinium (MPP^+^) in EpiAirway™ and Calu-3 monolayers. Cells were washed with Earle’s Balanced Salt Solution (EBSS) or Na^+^-free EBSS (-Na^+^) and incubated for the times indicated in the same buffer containing [^3^H]MPP^+^ (50 µM; 2 µCi/mL), either added to the apical or to the basolateral compartment, as indicated. The intracellular [MPP^+^] was determined as described in the Material and Methods Section. Points represent the mean ± SEM of three independent determinations.

The uptake of 1-methyl-4-phenylpyridinium (MPP^+^) has been initially measured at different times, up to 30 min, at both sides of EpiAirway™ (Figure 1). On the basolateral membrane, MPP^+^ uptake progressively increased in a time-dependent manner, following a linear trend. After 30 min, the intracellular concentration of MPP^+^ was about 150 µM, which is three times higher than the substrate concentration employed, i.e., 50 µM. In contrast, MPP^+^ influx at the apical side, although slightly increasing during the 30 min, was very low at any experimental time, hence excluding any significant accumulation of the substrate within the cells. As expected, the transport analysis performed in the absence of sodium confirmed that MPP^+^ uptake was completely independent from the presence of the cation. For comparison, the same time course analysis of MPP^+^ uptake has been reproduced in Calu-3 monolayers, showing that the transport was linear up to 30 min, sodium-independent, and concentrative at both the basolateral and the apical side.

**Figure 2:**
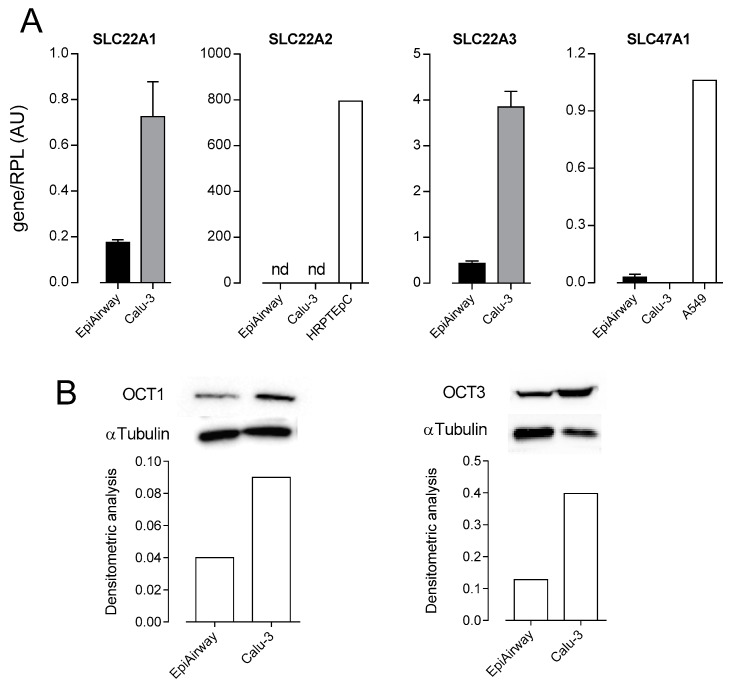
Expression of Organic Cation Transporters (OCTs) and Multidrug and Toxin Extruder 1 (MATE1) in EpiAirway™ and Calu-3. (**A**) mRNA levels for *SLC22A1*/OCT1, *SLC22A2*/OCT2, *SLC22A3*/OCT3, and *SLC47A1*/MATE1 were determined by means of qRT-PCR and normalized for the expression of the reference gene (*RPL15*) (see the Section 2). cDNA obtained from human renal proximal tubular epithelial cells (HRPTEpC) and A549 alveolar cells was employed as positive control for the analysis of *SLC22A2*/OCT2 and *SLC47A1*/MATE1 expression, respectively. Data are means ± SEM of five determinations. (**B**) The expression of OCT1 and OCT3 proteins was determined by Western Blot analysis, as described in the Material and Methods Section. Lower panels present the results of the densitometric analysis of protein expression, normalized to that of α-tubulin. A representative Western blot is shown, and the experiment was repeated two times, with similar results.

In order to identify the transporters responsible for MPP^+^ transport in respiratory cells, we next checked the expression of OCTs in EpiAirway™ and Calu-3 cells. In the same models, we also addressed the expression of *SLC47A1*/MATE1, because of its known interaction with MPP^+^ [5]. As shown in Figure 2, the mRNA for *SLC22A1*/OCT1 and *SLC22A3*/OCT3 were readily appreciable in both cell models, while *SLC22A2*/OCT2 was undetectable and *SLC47A1*/MATE1 was expressed at negligible levels. Protein bands corresponding to OCT1 and OCT3 were evident in both EpiAirway™ and Calu-3 cells, confirming the presence of these transporters in the models studied.

**Figure 3:**
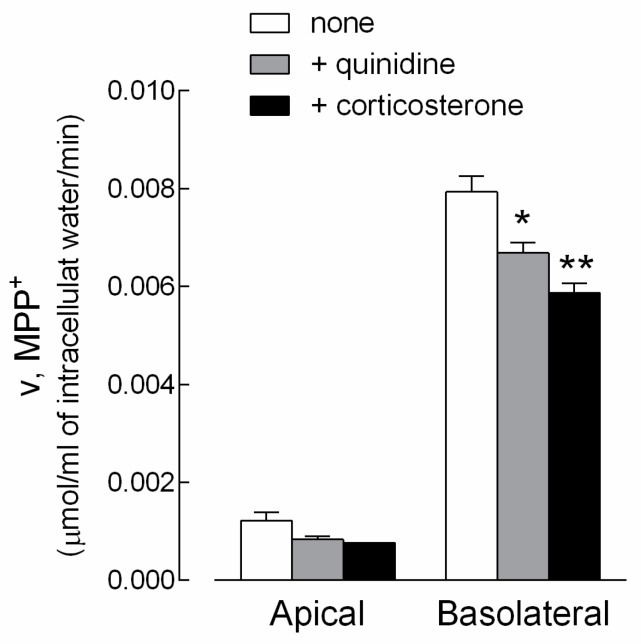
Characterization of MPP^+^ uptake in EpiAirway™. Monolayers of EpiAirway™ were washed with EBSS and incubated for 5 min in the same buffer containing [^3^H]MPP^+^ (50 µM; 2 µCi/mL), either added to the apical or to the basolateral compartment. Where indicated, quinidine (0.5 mM) or corticosterone (0.5 mM) were present during the transport assay. The intracellular (MPP^+^) was determined as described in the Material and Methods Section. Bars represent the mean ± SEM of three independent determinations. * *p* < 0.05; ** *p* < 0.01 versus none.

The actual contribution of OCT1 and OCT3 to MPP^+^ transport in EpiAirway™ was next addressed by measuring the uptake of the substrate in the presence of quinidine or corticosterone, which preferentially inhibit OCT1 [8,18,19] and OCT3 [5,20], respectively. The results, presented in Figure 3, indicate that both drugs were effective in inhibiting MPP^+^ influx at the basolateral side, thus pointing to the expression and activity of OCT1 and OCT3 on this side of cell cultures. In contrast, an only modest, not significant effect of the inhibitors was observed at the apical side. This finding, along with the low transport of MPP^+^ detected at this side (see Figure 1), excluded any relevant activity of the two carriers on the apical membrane.

**Figure 4:**
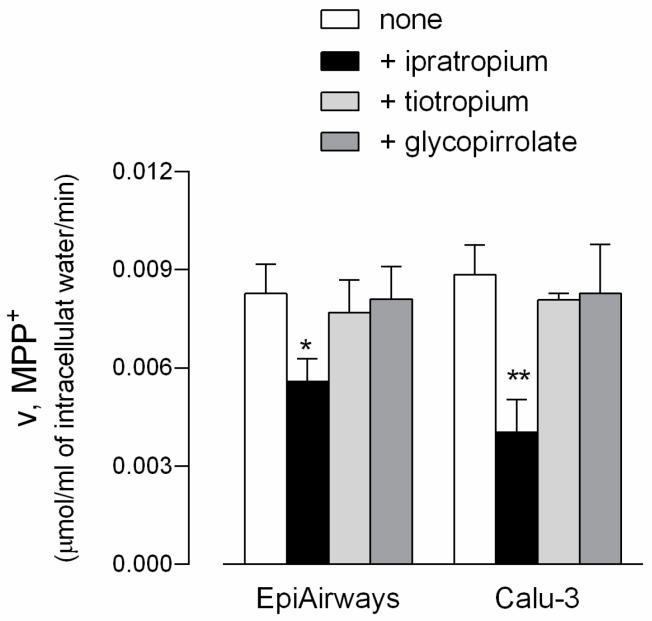
Effect of ipratropium, tiotropium, and glycopyrrolate on MPP^+^ uptake in EpiAirway™ and Calu-3 cells. Monolayers of EpiAirway™ and Calu-3 cells were washed with EBSS and incubated for 5 min, at the basolateral side, in EBSS containing [^3^H]MPP^+^ (50 µM; 2 µCi/mL) in the absence (none) or in the presence of 500 µM ipratropium, tiotropium, or glycopirrolate, as indicated. Bars represent the mean ± SEM of three independent determinations. * *p* < 0.05, ** *p* < 0.01 versus none.

In light of the recognized role of OCT transporters in drug delivery [26,27,28,29,30,31], the effect of three bronchodilators (i.e., ipratropium, tiotropium, and glycopyrrolate) on MPP^+^ uptake was next investigated in EpiAirway™, so as to examine the role of the transporters in the airways. Since these cells lack any transport activity on the apical membrane, the uptake was measured only at the basolateral side. As shown in Figure 4, ipratropium significantly reduced the basolateral uptake of MPP^+^ in both EpiAirway™ and Calu-3 monolayers, employed for comparison, while the two other drugs were ineffective in both cell models. This finding clearly points to an involvement of OCT1 and OCT3 transporters in the interaction with ipratropium, but not with tiotropium, nor glycopyrrolate. 

**Figure 5:**
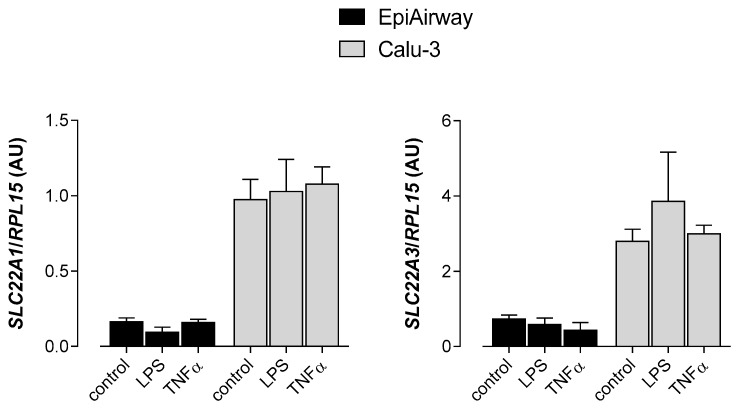
Effect of inflammatory stimuli on the expression of *SLC22A1* and *SLC22A3* in EpiAirway™ and Calu-3. LPS (10 µg/mL) and TNFα (10 ng/mL) were added to the culture medium for 24 h. The expression of *SLC22A1*/OCT1 and *SLC22A3*/OCT3 was determined with qRT-PCR and shown after normalization for the expression of the reference gene (*RPL15*). Data are means ± SEM of three independent determinations.

We then checked the possibility that inflammatory stimuli may modify the expression of OCT transporters, making them potential targets of anti-inflammatory drugs. To this end, we evaluated the expression of *SLC22A1*/OCT1 and *SLC22A3*/OCT3 in EpiAirway™ and Calu-3 cells incubated in the presence of the microbe-specific stimulus lipopolysaccharides (LPS) or of tumor necrosis factor α (TNFα). The results obtained, shown in Figure 5, indicate that pro-inflammatory conditions did not modify the mRNA level of *SLC22A1*/OCT1 nor *SLC22A3*/OCT3 in either cell model. 

**Figure 6:**
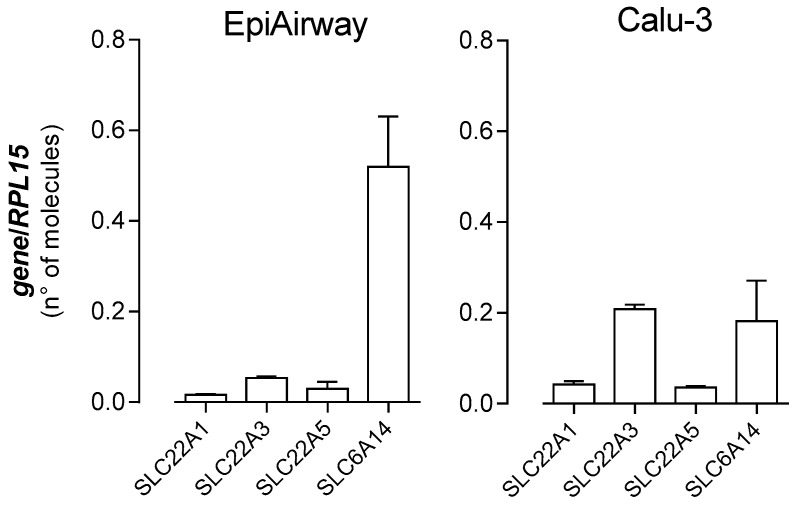
Expression of organic cation transporters in EpiAirway™ and Calu-3. The absolute quantification of the mRNAs for *SLC22A1*/OCT1, *SLC22A2*/OCT2, *SLC22A5*/OCTN2, and *SLC6A14*/ATB^0,+^ was performed as described in the Material and Methods Section by means of qRT-PCR. The expression of the gene of interest is expressed as number of molecules, upon normalization for the reference gene (*RPL15*). Data are means ± SEM of four determinations.

In a recent contribution, we have investigated the expression and activity of OCTNs in EpiAirway™ by addressing carnitine transport [32]. Results obtained have demonstrated the presence of OCTN2 on basolateral membrane, as well as the contribution of Na^+^-Cl^−^ cotransporter Amino acid Transporter B^0,+^ (ATB^0,+^) to carnitine uptake at the apical side. Therefore, in order to draw a complete picture of the expression of the transporters for organic cations in EpiAirway™, we quantified the amount of mRNA for OCT1 and OCT3 carriers, along with that of ATB^0,+^ and OCTN2 (Figure 6). As far as EpiArway™ are concerned, the most expressed mRNA is that for *SLC6A14*/ATB^0,+^, while the number of molecules of *SLC22A1*/OCT1, *SLC22A3*/OCT3, and *SLC22A5*/OCTN2, quantitatively comparable, are at least ten times less abundant. Calu-3, employed for comparison, expressed *SLC22A1*/OCT1 and *SLC22A5*/OCTN2 to the same extent as EpiAirway™. *SLC22A3*/OCT3 was, instead, four-fold, while *SLC6A14*/ATB^0,+^ was three times lower than in EpiAirway™.

## 4. Discussion

Many studies have, thus far, addressed the function of OCT transporters in different cell lines, employed as models of human pulmonary epithelium [13,14,15,16]. These reports highlighted a high heterogeneity among cell types in terms of transporters’ expression and activity; therefore, there is an increasing need for reliable models of normal bronchial epithelial cells to definitely define the expression and function of OCTs in the airways. To this end, in the present study, we employed EpiAirway™, a well-established in vitro model of polarized human bronchial epithelial cells that properly reflects the phenotype of respiratory barriers in vivo and is hence well-accepted for studies of pathophysiology, toxicology, inflammation, virus infection, and drug development [17].

Results obtained demonstrate that EpiAirway™ display an OCT transport activity which is confined to the basolateral compartment. The activity of both OCT1 and OCT3 is, indeed, readily detectable on the basolateral side of EpiAirway™, where the two transporters exert a concentrative uptake of MPP^+^, leading to a three-fold intracellular concentration of the substrate in 30 min. This finding is in line with immunofluorescent studies performed in human bronchi showing OCT3 in the entire plasma membrane of basal cells and on the basolateral membrane of intermediate cells [33]. In the same model, however, OCT1 was observed in the luminal membrane of ciliated epithelial cells. The basolateral localization of OCTs has also been observed in hepatocytes and kidney proximal tubule [9], where the transcellular movement of organic cations has been shown to depend upon the combined action of electrogenic OCT-mediated uptake at the basolateral side and the apical efflux supported by the pump *ABCB1*/P-gp and MATEs [5]. In our studies, however, the contribution of P-gp has been recently excluded in EpiAirway™ [34], while only a modest expression of MATE1 has been detected (see Figure 2).

No functional activity of OCTs has been observed at the apical side of EpiAirway™ cultures. This evidence is in contrast with what we previously reported for Calu-3 cells, where a clear-cut activity of OCT1 and OCT3 was measured on both the apical and the basolateral membranes of polarized monolayers [16]. Rather, at the apical side of EpiAirway™ cells, we have recently described a high activity of a transporter thus far not cited among the classical carriers for organic cations, i.e., *SLC6A14*/ATB^0,+^ [32]. This protein mediates a highly concentrative influx of substrates upon coupling with sodium and chloride [35], and besides transporting cationic and neutral amino acids, it also accepts carnitine [32]. Our results of mRNA quantitation clearly demonstrate that ATB^0,+^ is the most abundant transporter in EpiAirway™, being ten times more expressed than *SLC22A1*/OCT1, *SLC22A3*/OCT3, and *SLC22A5*/OCTN2. The role of this transporter in the lung seems to be related to an efficient protein clearance through the active reabsorption of amino acids, mainly under pathological conditions [36]. In particular, arginine has been shown to be taken up by ATB^0,+^ at the apical side of human airway cells and extruded toward the bloodstream through efflux systems y^+^L and b^0,+^ at the basolateral side [37]. The same transcellular pathways are employed by the inhibitors of nitric oxide synthase (NOS) N(G)-monomethylarginine (NMMA) and N-iminoethyl-lysine (NIL) [37], suggesting that this transporter may be involved in drug delivery in the airways. Consistently, we recently demonstrated that ATB^0,+^ interacts with bronchodilators glycopyrrolate and tiotropium in EpiAirway™ [32].

The role of OCTs and OCTNs in the uptake of drugs is, instead, well recognized, and their involvement in the intestinal absorption and renal excretion of many cationic drugs is well established [1,5,33]. They are known to mediate a highly concentrative uptake of a wide category of substrates, including classical anticancer molecules, such as platinum derivatives, and newly developed drugs for targeted therapy, such as imatinib and metformin [38]. In this context, the uptake is facilitated by the inside-negative plasma membrane potential, that allows to reach intracellular concentrations higher than outside the cells [39]. A role for OCTs/OCTNs in drug delivery has been also proposed in the airways, by demonstrating that the OCT-mediated uptake of 4-(4-(Diethylamino)styryl)-N-methylpyridinium iodide (ASP^+^) at the apical side of polarized Calu-3 cells is inhibited by bronchodilators formoterol, salbutamol, ipratropium, and the glucocorticoid budesonide [40]. Consistently, in the present contribution, we confirmed that the bronchodilator ipratropium, beside interacting with OCTN2 [32], also interacts with OCT1 and OCT3 in both Calu-3 cells and EpiAirway™.

In conclusion, our results indicate that EpiAirway™ lack any luminal activity of OCTs and OCTNs, and OCT1 and OCT3 are, indeed, active on the basolateral membrane and not at the apical side, where the major carrier involved in the transport of organic cation solutes is ATB^0,+^. The latter could thus be potentially listed, along with OCTs and OCTNs, among organic cation transporters responsible for drug delivery in the lung.

## Figures and Tables

**Table 1:** Sequences of the primer pairs employed for RT-qPCR analysis.

Gene/Protein Name NCBI Reference Sequence Identifier	Forward Primer	Reverse Primer
*SLC22A1* (NM_003057.3)	TGTCACCGAAAAGCTGAGCC	TCCGTGAACCACAGGTACATC
*SLC22A2*T2 (NM_003058.4)	CATCGTCACCGCGTTTAACCTG	AGCCGATACTCATAGAGCCAAT
*SLC22A3* (NM_003058.4)	AGGTATGGCAGGATCGTCATT	GCAGGAAGCGGAAGATCACA
*SLC22A5*/OCTN2 (NM_001308122.1)	TCCACCATTGTGACCGAG	ACCCACGAAGAACAAGGAGAT
*SLC6A14* (NM_007231.5)	CTGCTTGGTTTTGTTTCTTCTTGGTC	GCAATTAAAATGCCCCATCCAGCAC
*SLC47A1* (NM_018242.3)	TCAACCAGGGAATTGTACTGC	CAGAGCCTATCACCCCAAGA
*RPL15* (NM_001253379.2)	GCAGCCATCAGGTAAGCCAAG	AGCGGACCCTCAGAAGAAAGC
